# The Treatment of Asthma

**Published:** 1880-12

**Authors:** J. B. Berkart

**Affiliations:** Senior Assistant Physician to the City of London Hospital for Diseases of the Chest, etc.


THE TREATMENT OF ASTHMA. By J. B. BERKART, M.D., Senior Assistant Physician to the City of London Hospital for Diseases of the Chest, etc.

Not unfrequently the treatment of the paroxysms is of necessity limited to the exigencies of the moment; its sole aim, at the time, being the safe and speedy relief of the distressing symptoms. Although constantly attempted, this object has hitherto not been obtained, notwithstanding the long and tedious trials of all the means which theory and empiricism suggested for the purpose. The belief has consequently spread that unknown peculiarities—

caprices—of the disease are responsible for the failure of the treatment. But a glance at the subject will show that the want of success is really due to the indiscriminate application of remedies, of which each possesses a different physiological action. It will be readily conceded that chloroform and coffee, opium and stramonium, morphia and atropia, niter paper and emetics, tobacco and hot brandy and water, etc.—all side by side extolled as valuable remedial agents—are not so closely allied as regards their physiological actions as to be convertible; and if they are intended to break "the bronchial spasm"—which, I was amazed to learn, resists even chloroform (Thorowgood, Lettsomian Lectures, etc., 1879, page 72)—they require, each of them, special circumstances favorable for the production of that effect. But no such indications exist. The very idea of their existence is inconsistent with the prevalent theory of the disease. According to this, paroxysmal dyspnœa, accompanied by sibilation, denotes bronchial spasm, whatever else may be present at the time; and so significant are these symptoms, that attention to them permits to read off, as it were, the disease at a distance (Hyde Salter, *Lancet*, vol. i, 1870, page 147). The immediate consequence of this doctrine is that, in the choice of remedies—if choice there be—"no guide is known except"—the saddest of instructors—"the former experience of the patient" (Salter, *op. cit.*, page 183); hence a practice of which the following case is an apt illustration. "For 340 nights out of 365, the patient had to sit up, struggling for breath; and the effects of ordinary remedies may be thus briefly given: burning niter-paper, useless or worse than useless; tobacco or stramonium, smoked *ad nauseam*, slight benefit; chloroform inhalation, transient relief; nitrite of amyl, tried repeatedly and carefully, entirely useless; phosphorus, arsenic, iodide of potassium, no benefit whatever" (Thorowgood, *op. cit.*, p. 71). Such blind groping after remedies, in the presence of urgent symptoms, must needs defeat its own object. There can be no relief, safe and speedy—that is the lesson taught by failure—unless the remedies adopted are such as counteract or remove the proximate causes of the dyspnœa; and their selection depends in each case on the considerate appreciation of the surrounding circumstances.

It is not my present intention to enumerate all the measures that, in case of emergency, may be taken for the relief of a dyspnœal seizure. I propose merely to trace the indications for the purely medicinal treatment of those forms of asthma which are distinguished by the frequency of their occurrence and by the severity of the symptoms.

Foremost amongst them is œdema of the lungs, as it occurs in the obese and the cachectic, and in those suffering from valvular lesions of the heart, from gout, and from renal disease (uræmic asthma). It is invariably the result of a temporary failure of the left ventricle, while the right is still able to act;* and develops itself, either in the midst of apparently perfect health with the suddenness of a fainting fit, or as a rapid exacerbation of an existing cardiac derangement. To understand its pathology, it is well to remember that the constitutional and local causes just mentioned tend to impair the nutrition of the cardiac muscles to an extent varying from the cloudy swelling of the individual fiber, to its brown atrophy or fatty degeneration. The heart, notwithstanding these changes, continues, in ordinary circumstances, to perform its function in accordance with the requirements of the organism, and without painful perception by the patient. It is only when an increased demand is made upon its energy, and on the accession of an irritation, that the organ manifests its inherent weakness, by its inability to meet the one and resist the other, even if both are so slight as to be powerless to cause disturbance in a healthy person. Œdema is thus readily produced by imperfect ventilation of the lungs, as it arises from the rapid extension of bronchitis, from embolism of a large branch of the pulmonary artery and from extensive meteorismus. The reason is, that the blood, abnormally rich in carbonic acid, irritates the centers of respiration and circulation, and that, finally, while the right ventricle is able to empty part of its contents into the pulmonary artery, which possesses no tonus, the left is incapable of doing so, on account of the increased tension of the systemic vessels.

In these circumstances, the subcutaneous injection of one-sixth

---

* C. Mayer. *Bemerkungen zur Experimentelle Pathologie des Lungenœdems. Sitzungsber. der Akad. der Wissensch.* Wein, Band lxxvii. Abth. iii; Welch, *Zur Patholog. des Lungenœdems.* Virchow's *Archiv.* Band 72, Heft 2 and 3.

of a grain of morphia acts, indeed, like a charm. As soon as the morphia is absorbed, which requires a longer time than in health, the painful oppression at the chest and the hacking cough disappear; the noisy and frequent respiration becomes quiet and slower; the cyanosis of the face and lips give way to a flush; the cold and clammy skin becomes warm and moist; the contracted artery widens and fills; the heart regains its previous force and rythm, and with them return its impulse, its sounds, and its murmurs, while the consequences of its temporary failure as regards the lungs subside more or less completely. There is, subsequently, neither languor nor drowsiness, even in those who at other times are very susceptible to the influence of narcotics. Morphia merely counteracts the effect of the abnormal quantity of carbonic acid in the blood, and, with the attainment of that object, its influence is exhausted, as shown by the following cases:

Mrs. H., aged 42, tall, stout, has for several years been subject to cough and to attacks of dyspnœa. In the foggy weather of November, 1879, she was seized with smarting of the eyes; a great deal of sneezing, and running from the nose; an eruption of the upper lip; sore throat; harsh and painful cough, with scanty expectoration, and much dyspnœa; all the symptoms which, if they occur in summer, are supposed to form a special disease called hay-asthma. On Saturday, November 15th, she went to Eastbourne to get rid of her "cold," but, on arrival, all the symptoms, especially the dyspnœa, became much worse, and on Monday she had to return home. I saw her on November 17th, 1879, at 7 p.m., and noted the following. She sat in bed; she was cyanotic; there was great dyspnœa; she complained of pain across the chest and at the insertion of the diaphragm; there was a painful cough, with scanty expectoration; the extremities were cold. Pulse was very small, rapid, and almost countless; respiration noisy, frequent; there was a loud laryngeal noise. The chest wall was raised; the sterno-mastoids, were firmly contracted; the thyroid lying beneath the manubrium; the tongue was clean. On the right, front and back, the percussion note was flat; there was very feeble respiration above, and moist rhonchi at the base. On the left, front and back, better resonance, dry, and loud rhonchi from apex to base. The impulse of the heart was not felt; its area was enlarged to the left; sounds very feeble. Injection of morphia hydrochlorate, grain $\frac{1}{6}$. In ten minutes, perspiration commenced; the breath was easier, respiration deeper and slower, and the pain diminished. The laryngeal noise disappeared, the resonance of the right side improved, and inspiration was feebly heard; on the left, respiration returned, and the rhonchi became moist; the rhonchi at bases persisted; pulse improved; cardiac sounds distinctly heard. The patient was perspiring much when I left.

November 18th.—She passed a tolerably good night, but had no sleep. The cough was troublesome; the expectoration became more copious, and consisted of black mucous substance mixed with soot. A rational treatment of her bronchitis was now adopted, and she recovered by the end of the week.

Mr. L., aged sixty-two, tall, stout, for twenty years subject to gout, had signs of dilatation of the left ventricle, and of degeneration of the cardiac muscles. In December, 1878, he had an attack of gout in the left toe; subsequently, other joints were affected, and at last both lower extremities swelled. Bronchitis then followed, complicated by attacks of asthma. His friends noticed that he had frequent fainting fits: his face suddenly turned blue, the heart seemed to stop, and cold perspiration appeared on the forehead. On December 10th, 1878, when I saw him, he had great dyspnœa, with loud wheezing and troublesome cough. He felt exhausted from the many sleepless nights through which he had passed. Respiration rather frequent. The large and roomy thorax was generally deficient in resonance, and, on auscultation, there were loud, sonorous and sibilant rhonchi from apex or base. No cardiac impulse was seen or felt. The action of the heart was rapid, its sounds impure. The pulse was empty; there was considerable anasarca; the urine was scanty, but not albuminous; the tongue was thickly coated; great tympanitis; constipation. At 5 p. m., injection of hydrochlorate of morphia grain $\frac{1}{6}$. Immediate relief. He was ordered pil. hydrarg. gr. x. There was much improvement the next morning; the patient had passed a good night; he had no dyspnœa; the cough was less troublesome; and he had slept several hours. His bowels had copiously acted. He had a light breakfast, which he enjoyed. The expectoration consisted of a few pellets of black mucus. The thorax was much clearer. The pulse was felt at the wrist; it was frequent and intermittent. Now that the pressing symptoms of the moment had been successfully combated, a deliberate treatment was adopted, by which the patient greatly benefited.

In all cases of that kind, the subcutaneous injection of morphia is preferable to the internal administration of opium; for in the first place, the action of opium is not quite identical with that of its alkaloid; and, in the second, the absorption by the gastric mucous membrane is, owing to the stasis in the systemic vessels, slow and imperfect; so that, to obtain rapidly the desired effect, a very large dose would be required, of which the consequences would be still felt when the indication for the use of the drug had passed off. If, nevertheless, opium has to be administered internally, it should be given in a moderately large dose, and should be combined, to facilitate its absorption, with stimulants, as ether or aromatic spirit of ammonia.

But I must not omit to add that the subcutaneous injection of morphia is an operation with the performance of which neither the patient nor his friends may be entrusted. It is an operation, useful only in special circumstances, satisfying the imperative demands of the moment, but incapable of producing more than a transient benefit. Its thoughtless repetition with each recurrent attack not only not relieves—unless the dose be considerably increased—but aggravates the disease. A craving for morphia is readily established, but not easily eradicated.

To the use of stimulants in these cases the same objection applies as to that of opium. Their action, even in large doses, is comparatively slow; and, though they temporarily restore the activity of the heart, they do not remove so rapidly and so fully as morphia the peripheral obstacle—viz., the increased arterial tension. Dr. Thorowgood recommeds large doses of caffein—four grains in a cup of coffee (*loc. cit.*, p. 72). This preparation is, I presume, indicated if it be desired to produce copious diuresis, so as to diminish the volume of the blood; but, on account of the peculiar rigidity of the cardiac muscles which large doses of caffein are apt to produce, that remedy should be cautiously used.

The distressing form of dyspnœa which arises from extensive meteorismus requires for its relief the removal of the fermenting substances, either by emetics, if they are as yet in the stomach, or by enemta, if they are retained in the descending colon or rectum. If the removal be impracticable, the fermentation may be arrested by small (five-grain) doses of chloral-hydrate, or by about two drachms of the creasote mixture (*P. B.*), both of which—the former with the addition of a little mucilage—are given in some aromatic water. After one or two doses of these mixtures, the intense symptoms soon subside.

In cases of obstruction of the air-passages by plugs of mucus, the means of relief vary according to the position of the latter. Emetics are indicated if the block exist in the peripheral portions of the lungs; and, as they are employed only on account of the mechanical compression of the chest in the act of vomiting, preference is therefore to be given to those remedies which produce the least amount of depression. Hence the sulphate of copper or of zinc is preferable to antimony or ipecacuanha. I have frequently made subcutaneous injections of apomorphin—from one-twelfth to one tenth of a grain—and I have found them best answer the purpose.

Vomiting, i. e., compression of the chest, has little influence on the plugs of the larger bronchi. Hyde Salter correctly observed that, in the cases in which ipecacuanha afforded relief, this occurred before vomiting took place; and he truly remarked that ipecacuanha acted as a depressant—not, as he imagined, by relaxing the bronchial spasm, but by producing serous exudation around the tough pellets of mucus. The same effect, however, may be obtained in a less circuitous route, and with less general disturbance. The "antispasmodic fumes," which have been empirically recommended, owe their virtue to the presence of ammonia, and to that of picolin, of pyridin, of lutidin and collidin, as they are evolved in the combustion of niter-paper, of stramonium, of tobacco, and of the various patent "smoking mixtures." Ammonia and the members of the picolin series produce, when inhaled, intense hyperæmia, as may be seen in the buccal, pharyngeal and laryngeal mucous membranes of habitual smokers; and the exudation accompanying the hyperæmia tends to soften and to detach the obstructing mucus. Germain Sée has introduced for the purpose iodide of ethyl, of which from six to ten drops inhaled greatly increase the bronchial secretion. Its effect is transient, but unquestionably curative in cases of chronic bronchitis; whereas the fumes of stramonium and of similar drugs, especially if often repeated, aggravate the disease by the intense and lasting congestion which they produce.

The liquid extract of quebracho has of late been largely employed in the treatment of asthma. As yet, there is no indication for its use, except the presence of dyspnœa. A teaspoonful, repeated, if necesssary, at intervals of ten minutes, certainly relieves, as I have observed, the dyspnœa of phthisis, of pneumonia, of pleurisy, of emphysema, and of valvular lesions. It has failed of its effect, so far as I have seen, only in two cases of aortic disease; in the one, the patient had been for years accustomed to inhale nitrite of amyl almost every two hours; in the other, there was complication with marked attacks of stenokardia. The active principle of quebracho appears to be gum-resin; but as to its mode of action nothing is known. I have frequently noticed that, after the administration of the drug, there is slight flush of the face, perspiration and occasionally drowsiness; but there are no objective signs on the part of the heart and of the lungs sufficient to account for the relief.—*British Medical Journal.*

